# Isolation, characterization, and effectiveness of bacteriophage Pɸ-Bw-Ab against XDR *Acinetobacter baumannii* isolated from nosocomial burn wound infection

**DOI:** 10.22038/ijbms.2021.57772.12850

**Published:** 2021-09

**Authors:** Ladan Rahimzadeh Torabi, Monir Doudi, Nafiseh Sadat Naghavi, Ramesh Monajemi

**Affiliations:** 1 Department of Microbiology, Falavarjan Branch, Islamic Azad University, Falavarjan 84515/155, Isfahan, Iran; 2 Department of Biology, Falavarjan Branch, Islamic Azad University, Falavarjan 84515/155, Isfahan, Iran

**Keywords:** Acinetobacter baumannii, Bacteriophage, Burns, Drug Resistance, Lambda-Like Phage, Restriction Enzyme-Mapping, Sodium Dodecyl Sulfate-PAGE, Wound infections

## Abstract

**Objective(s):**

With emergence of drug resistance, novel approaches such as phage therapy for treatment of bacterial infections have received significant attention. The purpose of this study was to isolate and identify effective bacteriophages on extremely drug-resistant (XDR) bacteria isolated from burn wounds.

**Materials and Methods:**

Pathogenic bacteria were isolated from hospitalized patient wounds in specialized burn hospitals in Iran, and their identification was performed based on biochemical testing and sequencing of the gene encoding 16S rRNA. Bacteriophages were isolated from municipal sewage, Isfahan, Iran. The phage morphology was observed by TEM. After detection of the host range, adsorption rate, and one-step growth curve, the phage proteomics pattern and restriction enzyme digestion pattern were analyzed.

**Results:**

All isolates of bacteria were highly resistant to antibiotics. Among isolates, *Acinetobacter baumannii* strain IAU_FAL101 (GenBank accession number: MW845680), which was an XDR bacterium, showed significant sensitivity to phage Pɸ-Bw-Ab. TEM determined the phage belongs to *Siphoviridae*. They had double-stranded DNA. This phage showed the highest antibacterial effect at 15 ^°^C and pH 7. Analysis of the restriction enzyme digestion pattern showed Pɸ-Bw-Ab phage was sensitive to most of the used enzymes and based on SDS-PAGE, protein profiles were revealed 43 to 90 kDa.

**Conclusion:**

Considering the potential ability of the isolated phage, it had an antibacterial impact on other used bacterial spp and also strong antibacterial effects on XDR *A. baumannii.* Also, it had long latency and low burst size. This phage can be a suitable candidate for phage therapy.

## Introduction

Antibiotic resistance among bacterial infections has increasingly concerned both developed and developing communities and nations (1). The rapid spread of antibiotic-resistant bacteria has led to a serious challenge in the treatment of wound infections (2, 3). Burns are responsible for many pathophysiological changes (4) that lead to severe trauma (5, 6). Patients with burns are clearly at high risk for acquired infections which have been important causes of death in these patients (7, 8). Bacteria are the most frequent microorganisms responsible for wound infections. The frequency and diversity of bacterial infections in different wounds are influenced by factors such as wound type, depth, and area, the manner of wound formation, the amount of tissue loss, and the efficiency of host immune response (9). Wound colonization by pathogenic bacteria begins almost immediately after injury and the colonized bacteria involve opportunistic pathogens acquired from the environment or the patient’s microbial flora (10, 11). In many cases, only low-level microbial populations that grow in the wound impede wound healing (12). Among common gram-negative bacteria in burn wounds, the drug-resistant *Pseudomonas aeruginosa*, *Acinetobacter baumannii*, members of Enterobacteriaceae, and other broad-spectrum Beta-lactam-producing bacteria have special importance (13, 14). *A. baumannii* is recently detected among the most abundant nosocomial wound infecting bacteria, eliminating of which from hospital wards remains a serious challenge due to its long survival time in the hospital environment (15). On the other hand, the increasing pattern of antimicrobial resistance among *A. baumannii* strains has caused a great deal of concern (16, 18). *A. baumannii, Escherichia coli*, and *Klebsiella pneumoniae* are gram-negative bacteria and are some of the opportunistic and nosocomial pathogens of hospital-acquired infections in burn patients (19, 20). Phages are viruses that infect bacteria and are used as antibacterial agents in the treatment of pathogenic and infectious bacteria. Recognition of effective phages can significantly solve the problems due to the emergence of increasing drug-resistant infectious pathogens (21-23). Some burn wounds related to phage therapy research (24-26) offere that phages could have the ability to limit bacterial wound infections in burn patients. Also, multiple pieces of research have illustrated the advantages of phage therapy to inhibit MDR bacterial infections in burn patients (27, 28). Lavergne *et al.* studied the alternative treatments for multidrug-resistant bacterial infections. They evaluated a case of bacteriophage-treated multidrug-resistant *A. baumannii* infection and concluded that determination of the exact role of phages as a chance for inhibiting multidrug-resistant bacterial infections needs more clinical trials (29). These conclusions were also obtained by Yang *et al*. and Jin *et al*. (30, 31). Researchers isolated the bacteriophages that were effective against MDR bacterial isolates from septic ulcer infections. Phage PA DP4 was effective on *P. aeruginosa*, phage KP DP1 was effective on *K. pneumonia* and phage EC DP3 was effective on *E. coli*. Moreover, the results of that study also showed that phages can be a valuable choice for prophylaxis against septic ulcers (32). Furthermore, researchers designed a new lytic bacteriophage cocktail with therapeutic potential against bacteria that had caused diabetic foot infections (33). Researchers examined the stability of bacteriophages in burn wound care products. According to the results of their study, supplementation of bacteriophages in burn wound care products led to increased rate of antimicrobial activity. However, some topical antimicrobial compounds traditionally used to prevent and treat burn wound infections may decrease phage activity (34). As regards some queries regarding MDR, XDR, and PDR bacterial infection treatment being unresolved in burn patients, other ways must be tried. Here, we reported both morphological identification of phage targeting resistant MDR and XDR *A. baumannii*,* K. pneumonia *and* E. coli* isolated from burn wounds and characterization of phage survival, host range, adsorption rate, one-step growth curve, proteomics pattern, and restriction enzyme mapping where we used some endonuclease enzymes for digestion analysis which were not mentioned in other articles.

## Materials and Methods

***Isolation and biochemical identification of bacterial strains***

In this study, 50 patients with burn wounds were randomly selected in several burn-specialized hospitals in different areas of Iran. Patients’ wounds were sampled over a period of three months (March to May 2020). Bacterial isolates causing infections were isolated from various burn wounds. Using morphological and biochemical tests, the initial identification of the bacteria was performed. The biochemical tests included IMVIC, TSI, MR, VP, motility, H_2_S and Indole production, OF, catalase, oxidase, urease, nitrate reduction, and citrate utilization (Merck, Germany) (35, 36). Standard strains of *K. pneumoniae* ATCC10031, *E. coli* ATCC25922, and *A. baumannii* 19606 ATCC were prepared as lyophilized ampoules from Pasture Institute of Iran.

***Molecular identification of bacterial isolates***

Bacterial DNA was extracted using a nucleic acid extraction kit (RIBO-prep, Russia) according to the manufacturer’s protocol. Molecular identification of the bacterial isolates was performed based on the 16S rRNA gene sequence. For this purpose, the pair of universal primers: 1492R (5′ACGGCTACCTTGTTACGACTT3′) and 27F (5′AGAGTTTGATCCTGGCTCAG3′) prepared by (Pishgam Co. Iran) under license from Metabione Co. of Germany were used. The PCR reaction was performed in a thermal cycler (T100 Bio-Rad, Malaysia). The sequences of all PCR products were then determined in a DNA sequencer (3130, Applied Biosystems) ​​according to the manufacturer’s instructions (37-39).

***Antibiotic resistance pattern testing***

To evaluate the antibiotic resistance pattern in gram-negative clinical isolates, an antibiogram test was performed by the agar disk diffusion method. The diameter of growth inhibition zones was measured following incubation at 37 ^°^C for 24 hr. The XDR isolates had the highest resistance and lowest susceptibility to the tested antibiotics. The antibiogram discs (CONDA, Spain) were selected according to CLSI standard reference because extensively drug resistant (XDR) means non-susceptible to more than one agent in all but two or fewer antibiotic classes and multi drug resistant (MDR) means non-susceptible to more than one factor in three or more antimicrobial classes (40, 41).

***Isolation and enrichment of bacteriophage***

Municipal inlet sewage was used to isolate possible phages. Sampling was done from the northern and southern municipal sewage treatment plants of Isfahan in Iran. The samples were then transferred to the laboratory on ice. Each sample was centrifuged at 8000g for 15 min at 4 ^°^C. Then, 10 ml of supernatant was filtered through a 0.22 μm diameter syringe filter and the filtered solution was added to 50 ml of 2×BHIB (Ibresco, Iran). Then, 100 μl of each fresh overnight bacterial culture was separately added to each medium and the mixtures were shaken overnight at 140 rpm, 37 ^°^C (42).

***Enumeration of the isolated bacteriophage particles***

The double-layered agar plate technique (overlay method) was used for plaque formation. For this purpose, serial dilutions of each phage filtrate solution (10^8^=PFU/ml) was prepared in 50 ml SM buffer (1 M Tris-HCl pH 7.5, 5.8 g NaCl, 2g MgSO_4. _6H_2_O in 1 L distilled water) and 0.1 ml of each dilution was separately added to 0.1 ml of fresh bacterial culture (10^8^ CFU/ml). Finally, this mixture was added to 5 ml of melted BHI medium containing 0.7% agar, and the mixture was added to the surface of BHI with 1.5% agar, the prepared culture was then incubated for 24 hr at 37 ^°^C. Afterward, the formed plaques were observed and counted (23, 43).

***Phage plaques purification and bacteriophage serial dilution***

In order to purify specific phage, overlay agar plates were prepared with 10^-4^ to 10^-8^ dilutions of supernatants containing phage. The single plaques formed on the plate surface were cut with a sterile scalpel blade and transferred to sterile microtubes containing 1 ml SM buffer and mixed thoroughly for 30 sec. The mixtures were then centrifuged at 8000 g for 5 min at 4 ^°^C, and then 0.1 ml of each separated supernatant was added to 0.9 ml of SM buffer. Plaque formation by each solution was investigated using the overlay method, and the plaques were counted after incubation for 24 hr at 37 ^°^C (44).

***Assessment of bacteriophage host range***

The host range of the studied phage was investigated using the spot method on different strains of bacteria. Accordingly, 0.1 ml of fresh overnight bacterial culture (1.5×10^8^ CFU/ml) was mixed with 5 ml of melted BHI medium containing 0.7% agar and poured on the surface of BHI with 1.5% agar. After solidifying of the media, 10 μl of each dilution prepared from phage filtrates in the SM buffer was inoculated as spots on the agar surface in separated areas. After 24 hr incubation at 37 ^°^C, the spot test results were reported as «++» for complete lysis, «+» for opaque or weak lysis, and «-» for non-lysis (45, 33).

***Morphology and structure of bacteriophage by TEM***

For a detailed study of the structure and morphology of possible bacteriophages, 10 μl of phage filtrate (10^8 ^PFU/ml) was carbon coated on a copper grid for 30 sec and stained with 2% uranyl acetate (w/v) for 1 min. After drying, phage particles were observed in the sample with TEM (EM 208S 100 Kv, Philips). Detection of the phage family according to the morphological features was performed by using the latest changes in the International Virus Classification Committee (ICTV) reports (46-48, 43).

***Appraisement of bacteriophage adsorption rate***

The mixture containing equal volumes of bacterial strain fresh culture (1.5×10^8^ CFU/ml) and phage stock (10^6^ PFU/ml) was prepared incubated at 37 ^°^C. Then, the host phage mixture was centrifuged at different time intervals (0, 5, 10, 15, 20, 25, and 30 min) at 8000 g to precipitate phage-absorbing cells. Then the titers of unabsorbed phages in the supernatant were determined by the overlay method (43).

***Phage stability in the presence of different pH and temperatures***

To measure the viability of isolated phages at different pH values, the pH of the SM buffer was adjusted in the range of 4-10 and then the phage filtrate (10^8 ^PFU/ml) was added to the buffer with each pH value. After incubation for 1 hr at 37 ^°^C, the survival rate of the phage was determined by counting the titers of active phages using the overlay method. In order to investigate the antibacterial effect of phages at different temperatures, 10 μl of each phage filtrate solution (10^4^-10^8 ^PFU/ml) was added to overlayed bacterial cultures, then incubated at various temperatures (15, 20, 30, 37, and 42 ^°^C). The temperature at which clear zones were observed, was determined as the optimum temperature for the antibacterial activity of the isolated phage (43).

***Obtaining the desired multiplicity of infection (MOI)***

The proportion of plaque-forming unit (PFU) to colony-forming unit (CFU) was considered as MOI. For this purpose, different dilutions of 10^5^ to10^7^ PFU/ml were mixed separately with 10^6^ CFU/ml of bacteria. The numbers 10^5^, 10^6^, and 10^7 ^PFU/ml were considered for MOI=10, MOI=1, and MOI=0.1, respectively. For MOI 0, phage-free bacteria were considered as bacterial growth control. Then the plate was shaken at 37 ^°^C for 13 hr and the light absorption of the wells was read continuously every 1 hr at OD=600 nm and the results were recorded (49, 50). 

***One-step phage growth curve plotting***

To obtain the latency period and the size of the phage burst size, first 1 ml of bacterial culture with OD=0.2 at the wavelength of 600 nm in BHI broth was added to different dilutions of the isolated phage and incubated at 37 ^°^C. After 6 min of incubation, the culture was centrifuged at 6000 g for 10 min at 4 ^°^C to remove residual phage particles. The resulting precipitate was then added to 50 ml of BHI broth medium and re-incubated at 37 ^°^C. Then, every ten min, the virus titer was determined using the overlay method in terms of PFU/ml, and the resulting curve was plotted (51, 52).

***Extraction of bacteriophage genomic DNA***

First, the enriched phage stock was treated with 1 µg/ml *DNase I* and *RNase A* enzymes (Fermentas/Thermo Fisher Scientific, USA) for 20 min at 37 ^°^C, and then passed through 0.22 μm syringe filters and were centrifuged at 28000 g for one hour. Phage DNA extraction was performed using a genomic DNA extraction kit (NORGEN Biotek, Canada). To do this, a titer above 10^8 ^PFU/ml of phage particles an equal volume of phenol/chloroform/isoamyl alcohol (25/24/1) was added to remove protein residues. Phage DNA was then precipitated by isopropanol in the aqueous phase and washed twice with ethanol (53).

***Sensitivity assessment by digestion profile***

The purified nucleic acid of phage was examined for its sensibility versus* HindIII, EcoRI, BamHI, KpnI, HaeIII, *and* XhoI* enzymes (Fermentas /Thermo Fisher Scientific, USA). These enzymes were used to analyze the digestion pattern. For this purpose, the phage DNA was blended with every endonuclease enzyme and incubated at 37 ^°^C for 16 hr. The results were analyzed by 1% agarose gel electrophoresis at 75 V (43).

 ***Appraisement of bacteriophage proteomics pattern***

The phage particles which were purified in CsCl were examined in SDS-PAGE 10%. For this purpose, 20 ml of each sample of phage filtrate was mixed with 5 μl SDS PAGE sample 6X loading buffer (50 mM Tris HCl [pH=6.8], 2% (w/v) SDS, 10% (w/v) glycerol, 5% (v/v)2-mercaptoethanol, and 0.001% (w/v) bromophenol blue, 4.7 ml distilled water) and then heated in boiling water for ten minutes. Separated bands created by phage proteins were stained and visualized in the gel by using Coomassie Brilliant Blue dye (45).

***Statistical analysis***

The results were presented as SEM±Mean. One-way ANOVA and GraphPad Prism 8.0 software were used to analyze the data and to compare the experimental groups. SPSS version 20 was used for analytical statistics.

**Figure 1:**
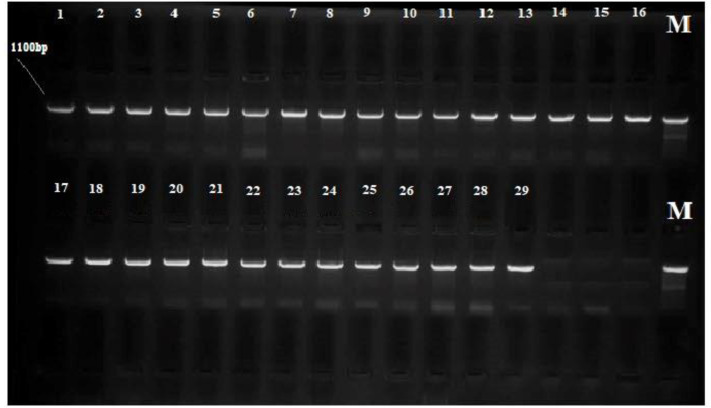
The amplified fragments obtained from PCR for 16S rRNA gene of *Acinetobacter baumannii* (1-16), *Klebsiella pneumonia* (17-21), and *Echerichia coli* (22-29) isolated from burn wounds, the optimum size of *PCR* fragments for detection was 1100 bp; M: Marker (100–3000 bp)

**Table 1:** Preliminary identification of the bacteria isolated from burn wounds

*Klebsiella pneumonia*	*Escherichia coli*	*Acinetobacter baumannii*	**Tests**
**Negative**	Negative	Negative	Gram staining
**Rods**	Rods	CoccoBacilli	Morphology
**A/A/-G**	A/A + G	K/K –H_2_S	TSI
**+**	-	-/+	Urease
**Fermentative**	Fermentative	Oxidative	OF
**Non-Motile**	Motile	Non-Motile	Motility
**+**	-	-	VP
**-**	+	-	MR
**-**	-	-	Oxidase
**+**	+	+	Catalase
**+**	-	+	Citrate
**-**	+	-	Indole
**+**	+	-	Nitrate reduction
**+**	+	-	ONPG test
**+**	+	+	Glucose fermentation
**+**	+	+	Xylose fermentation
**+**	+	+	Lactose fermentation
**+**	+	-	Maltose fermentation
**+**	+	+/-	Mannitol fermentation
**-**	-	-	Coagulase
**-**	-	-	Gelatin Hydrolysis
**-**	-	-	Spore formation

-: Negative results; +: Positive results; +/-: Variable results; OF: Oxidative-Fermentative; VP: Voges-Proskauer; MR: Methyl Red; TSI: Triple Sugar Iron; OPNG: O-Nitrophenyl-β-D-Galactopyranoside; K: Alkaline; A: Acid; G: Gas produced; H_2_S: hydrogen sulfide produced

**Table 2:** Characteristics of hospitalized patients with burn wounds in this study

Signs	N. Patients (%)	
		**Patient ages (years)**
	4 (13.8%)	10-20
	6 (20.7%)	21-30
	10 (34.5%)	31-40
	5 (17.20%)	41-50
	4 (13.8%)	˃51
		**Gender**
	17 (58.62%)	Male
	12 (41.38%)	Female
		**Burn degree**
**Red, painful, no blisters**	10 (34.48%)	First/degree burns
**Red, blistered, swollen, and painful**	8 (27.59%)	Second/degree (partial) burns
**Underlying bones and muscles**	11 (37.93%)	Third/degree (deep) burns
		**Reasons for the burn**
	12 (41.38%)	**Thermal**
	9 (31.03%)	Chemical
	2 (6.90%)	Radiation
	5 (17.24%)	Electrical
	1 (3.45%)	Extreme sun exposure
		**Hospital stay (weeks)**
	6 (20.7%)	˂1
	14 (48.27%)	1-3
	9 (31.03%)	˃3
		**Burn Zones**
	4 (13.8%)	Head and neck
	3 (10.34%)	Trunk and spine
	9 (31.03%)	Upper extremities
	2 (6.90%)	Genital organs
	11 (37.93%)	Lower extremities

Etiology distribution of severe burn patients by age, gender, degrees, signs, reasons, the length of hospitalization, and burn sites graphically represented. The number and percent of burn patients among the total number of isolates (N. Patients (%)) are shown

**Table 3:** Results from antimicrobial resistance experiment of G-negative bacteria isolated from burn wounds

*Klebsiella pneumonia* (N = 5)		*Escherichia coli* (N = 8)		*Acinetobacter baumannii* (N = 16)		Antibiotics
**R (%)**	R count	R (%)	R count	R (%)	R count	
					
**100**	5	87.5	7	100	16	Aztreonam
**60**	3	75	6	87.5	14	Cefoxitin
**80**	4	37.5	3	68.75	11	Cloxacillin
**80**	4	37.5	3	87.5	14	Clindamycin
**100**	5	75	6	93.75	15	Co-amoxiclav
**60**	3	62.5	5	56.25	9	Polymixin B
**100**	5	50	4	87.5	14	Ciprofloxacin
**60**	3	50	4	62.5	10	Imipenem
**100**	5	25	2	93.75	15	(Pipracillin-tazobactam) Tazocin
**60**	3	50	4	100	16	Penicillin G
**100**	5	75	6	87.5	14	Tobramycin
**80**	4	50	4	81.25	13	Amoxicillin
**100**	5	87.5	7	75	12	Co-Trimoxazole
**80**	4	75	6	87.5	14	Cefepime
**80**	4	87.5	7	62.5	10	Cefotaxime
**100**	5	75	6	87.5	14	Meropenem
**60**	3	62.5	5	81.25	13	Ceftazidime
**100**	5	37.5	3	93.75	15	Gentamycin
**80**	4	75	6	75	12	Cefixime
**100**	5	87.5	7	50	8	Ampicillin-Sulbactam
**40**	2	25	2	37.5	6	Colistin
**100**	5	75	6	100	16	Tetracycline
**60**	3	62.5	5	93.75	15	Amikacin
**60**	3	37.5	3	100	16	Ceftriaxone

The number and percent of resistant (R) isolates among the total number of isolates (N) are shown; in this study of a total of 29 isolates, *Acinetobacter baumannii* and *Klebsiella* *pneumonia* were the most resistant isolate and identified as XDR: Extensively drug-resistant (non-susceptibility to minimum one factor in all but two or fewer antibiotic classes/resistant to all antibiotics except colisitin in this study); *Echerichia coli* identified as MDR: Multi-drug-resistant (non-susceptibility to minimum one factor in three or more antibiotic classes); Overall Gram-negative isolates were found to show maximum susceptibility to colistin

**Figure 2:**
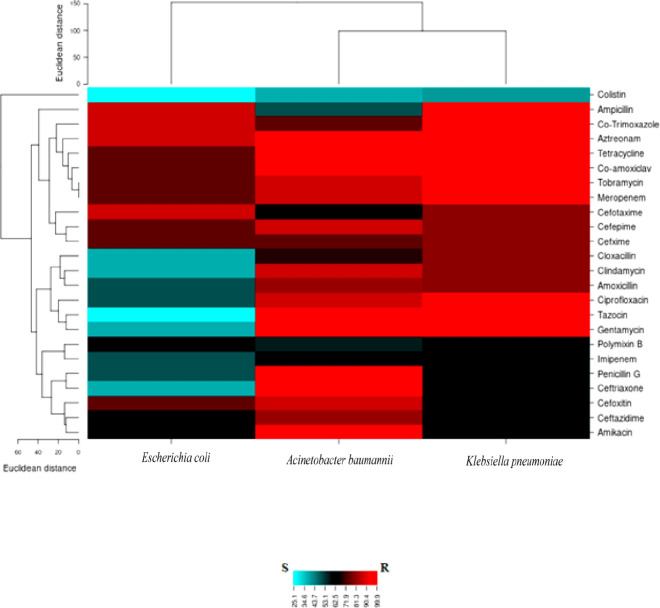
Heat map results from antimicrobial resistance patterns for *Acinetobacter baumannii*, *Klebsiella pneumonia*, and *Echerichia coli* isolated from a hospitalized patient with burn wounds; R:Resistance (Red regions); S:Susceptible (Blue regions); Intermediate (Black regions)

**Figure 3:**
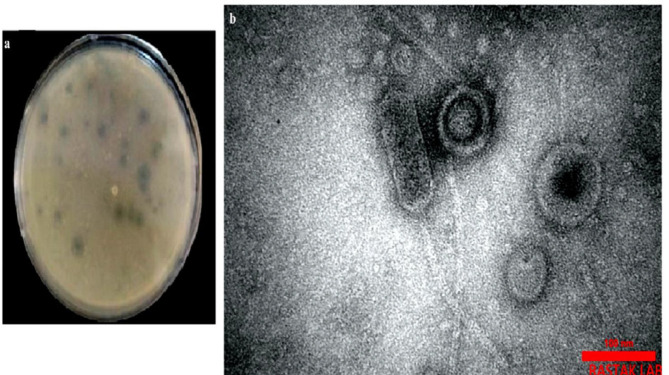
Primary plaques appearance of isolated phage(a); TEM micrograph of the lytic phage of the *Acinetobacter baumanni*i strain IAU_FAL101isolated from municipal sewage, Isfahan, Iran related to the family *Siphoviridae*, Phage tail length was estimated 160±10 nm and the phage head diameter was estimated 100 nm (b); Scale bars show 100 nm

**Table 4:** Host range of the lytic phage Pɸ-Bw-Ab on gram-negative bacteria isolated from burn wounds

Lab isolate ID	Pathogen	Spot test	Source	Area of isolation (cities in Iran)
**Shmy01**	*Acinetobacter baumannii*	-	Shohada Mehrab Hospital (Burn)	Yazd
**Shmy02**	*Acinetobacter baumannii*	-	Shohada Mehrab Hospital (Burn)	Yazd
**Shmy03**	*Acinetobacter baumannii*	++	Shohada Mehrab Hospital (Burn)	Yazd
**Shmy05**	*Escherichia coli*	-	Shohada Mehrab Hospital (Burn)	Yazd
**Shmy06**	*Acinetobacter baumannii*	+	Shohada Mehrab Hospital (Burn)	Yazd
**Shmy07**	*Escherichia coli*	-	Shohada Mehrab Hospital (Burn)	Yazd
**TA05**	*Klebsiella pneumonia*	-	Taleghani Burn Injuries Hospital	Ahvaz
**TA07**	*Acinetobacter baumannii*	-	Taleghani Burn Injuries Hospital	Ahvaz
**TA08**	*Acinetobacter baumannii*	-	Taleghani Burn Injuries Hospital	Ahvaz
**IMK01**	*Acinetobacter baumannii*	-	Imam Musa Kazim Hospital	Isfahan
**IMK03**	*Acinetobacter baumannii*	-	Imam Musa Kazim Hospital	Isfahan
**IMK07**	*Escherichia coli*	-	Imam Musa Kazim Hospital	Isfahan
**IMK09**	*Acinetobacter baumannii*	-	Imam Musa Kazim Hospital	Isfahan
**IMK10**	*Escherichia coli*	-	Imam Musa Kazim Hospital	Isfahan
**IMK11**	*Acinetobacter baumannii*	+	Imam Musa Kazim Hospital	Isfahan
**IMK12**	*Acinetobacter baumannii*	+	Imam Musa Kazim Hospital	Isfahan
**TM01**	*Acinetobacter baumannii*	-	Mottahari Burns Hospital	Tehran
**TM02**	*Escherichia coli*	-	Mottahari Burns Hospital	Tehran
**TM03**	*Klebsiella pneumoniae*	-	Mottahari Burns Hospital	Tehran
**TM04**	*Escherichia coli*	-	Mottahari Burns Hospital	Tehran
**TM05**	*Klebsiella pneumoniae*	-	Mottahari Burns Hospital	Tehran
**TM06**	*Escherichia coli*	-	Mottahari Burns Hospital	Tehran
**TM07**	*Klebsiella pneumoniae*	-	Mottahari Burns Hospital	Tehran
**VRC01**	*Acinetobacter baumannii*	-	Velayat Burn Injuries Hospital	Rasht
**VRC02**	*Acinetobacter baumannii*	+	Velayat Burn Injuries Hospital	Rasht
**VRC06**	*Escherichia coli*	-	Velayat Burn Injuries Hospital	Rasht
**VRC09**	*Acinetobacter baumannii*	-	Velayat Burn Injuries Hospital	Rasht
**VRC10**	*Klebsiella pneumoniae*	-	Velayat Burn Injuries Hospital	Rasht
**ATCC 25922**	*Escherichia coli*	-	ATCC	Pasteur Institute of Iran
**ATCC 19606**	*Acinetobacter baumannii*	-	ATCC	Pasteur Institute of Iran
**ATCC 10031**	*Klebsiella pneumonia*	-	ATCC	Pasteur Institute of Iran

- : Without effect; + : Opaque and weak zone; ++: Transparent zone. Ahvaz Ayatollah Taleghani Burn Hospital (TA); Isfahan Imam Musa Kazem Burn Hospital (IMK); Tehran Shahid Mottahari Burn Hospital (TM); Rasht Velayat Educational Medical Center, Burn Hospital (VRC); Yazd Shohada Mehrab Hospital (Shmy), Iran. Lab isolate IDs except ATCC are our clinical isolates (laboratory collection); ATCC(American Type Culture Collection (from Pasteur Institute of Iran

**Figure 4:**
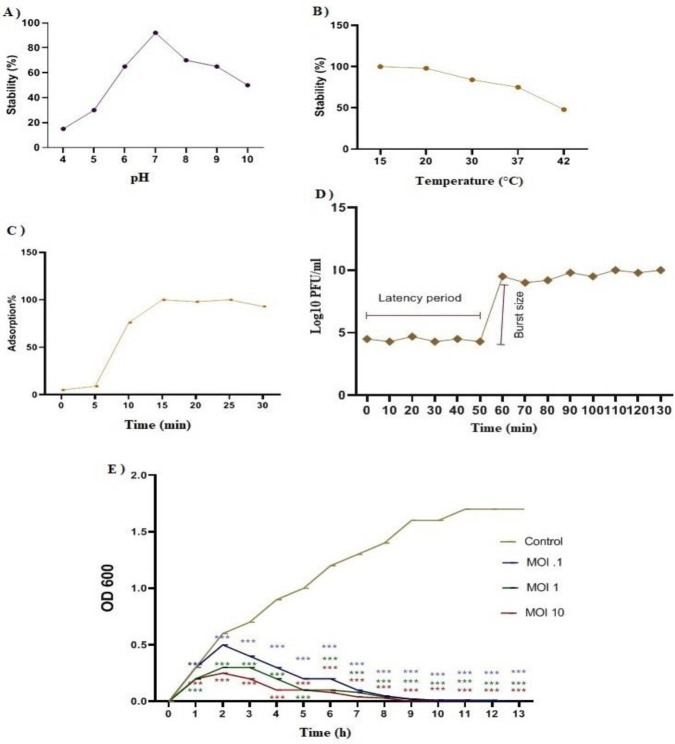
pH stability of bacteriophage Pɸ-Bw-Ab incubated for 60 min under pH range (4-10) (*P<*0.01) (A). Thermal stability of Pɸ-Bw-Ab bacteriophage incubated for 60 min under several temperatures (*P<*0.01) (B). Adsorption procedure of Pɸ-Bw-Ab (*P<*0.01) (C). *The mean±SEM of triple experiments. One-way ANOVA analysis of variance showed a significant difference in results from different conditions (*P<*0.01). One-step growth curve of the phage Pɸ-Bw-Ab on *Acinetobacter baumannii* strain IAU_FAL101.*One-way ANOVA analysis of variance displayed that a significant difference was observed in the chart (*P<*0.001) (D). Bacterial cell lysis by the phage Pɸ-Bw-Ab at several MOI of 0.1, 1, and 10 on *Acinetobacter baumannii* strain IAU_FAL101 according to optical density in 600 nm. *One-way ANOVA analysis of variance showed that there was a significant difference between different groups (*P<*0.001) (E). * Every value indicates the means of three mean±SD

**Figure 5:**
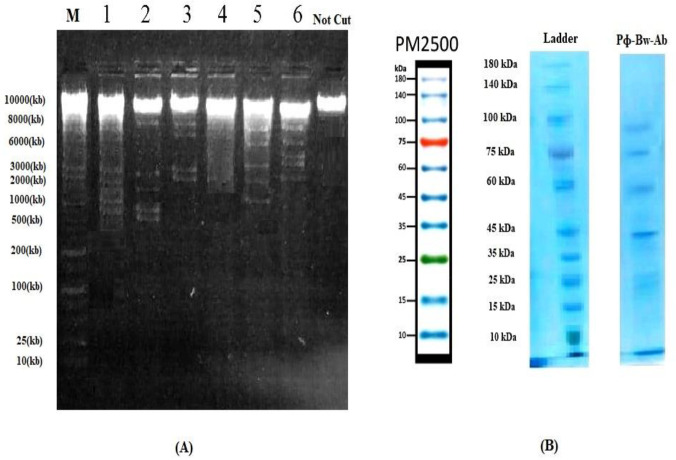
Restriction map of the phage Pɸ-Bw-Ab digested with restriction enzymes *HindIII* (tier 1), *EcoRI *(tier 2), *XhoI* (tier 3), *HaeIII* (tier 4), *Bam HI* (tier 5), and *KpnI* (tier 6); Lane M:Ladder or Marker, 25 Kb DNA marker (Fermentas/Thermo Fisher Scientific, USA) (A); The proteomic patterns of Pɸ-Bw-Ab on a 10% SDS-PAGE according to 10 to 180 kDa of ladder (B)

## Results

***Biochemical and molecular identification of the bacterial isolates***

A total of 29 bacterial isolates from burn wounds of the studied patients were identified and confirmed by biochemical and molecular tests. Among the hospitalized patients, 29 had gram-negative bacterial infections. The bacterial characteristics by biochemical tests were confirmed. *A. baumannii*, *E. coli*, and *K. pneumonia* were the most common gram-negative bacteria causing burn wound infection in this study. The results from BLAST analysis of the amplified fragments in 16S rRNA gene sequencing of the isolated bacteria showed that the bacteria were strains of *A. baumannii*,* K. pneumonia, *and* E. coli *with 98–99% identity (Figure 1) (Table 1).

***Characteristics of the patients with burn wounds***

In this study, 29 patients were eligible and had antibiotic-resistant burn wound infections. According to descriptive statistics, most of the patients were male (58.62%) and the patients aged 31 to 40 years had the highest population (34.5%). Among hospitalized patients, 37.93% had third-degree burns. The most common cause of burns was explosion (41.38%) followed by chemical burns (31.03%). The highest length of hospitalization was 1 to 3 weeks (48.27%). The upper limbs of burn patients had more involvement (37.93%) than other limbs (Table 2).

***Evaluation of antibiotic resistance pattern in hospitalized burn patients***

Antibiotic resistance pattern obtained from agar disk diffusion method in clinical isolates showed that among the isolates, *A. baumannii* strain IAU_FAL101 (GenBank accession number: MW845680) that called Shmy03 showed above 80% to 100% resistance to most antibiotics. The lowest resistance of the bacterium was observed to the antibiotic colicitin (37.5%). *E. coli* isolates showed the highest resistance to aztreonam, cotrimoxazole, and ampicillin-sulbactam (87.5%), and the lowest resistance was seen to colicitin (25%). *K. pneumoniae* isolates were 80% to 100% resistant to most antibiotics and 40% resistant to colicitin (Table 3). According to the results, *A. baumannii* and *K. pneumoniae* were detected as XDR. Heat map results from antimicrobial resistance testing were shown in Figure 2. 

***Enumeration of isolated bacteriophage***

The clear plaques were observed after the spotting test of phage filtrate on the BHI agar culture of *A. baumannii* strain IAU_FAL101. In the BHI agar medium, 31 PFU was counted on a plate and the total titer of *A. baumannii* strain IAU_FAL101 lytic phages was approximately detected as 31×10^8^ PFU/ml (Figure 3A). 

***The morphology evaluation by TEM***

The lytic phages were isolated from the raw inlet sewage of Isfahan treatment plants in Iran by the double-layer method. The isolated lytic phages Pɸ-Bw-Ab, which formed clear plaques on the bacterial culture, were morphologically determined by TEM, and further studies were done on it. Observation of the morphology of the phage by TEM showed that this phage was a dsDNA virus with a narrow long tail and a cylindrical shape belonging to the family *Siphoviridae *among the order *Caudovirales*. Phage tail length was estimated at 160±10 nm and the phage head diameter was estimated to be 100 nm (Figure 3B).

***The host range designation***

The results from host range and lytic activity of the phage Pɸ-Bw-Ab on different clinical strains showed that among different bacterial strains, *A. baumannii* strain Shmy03 named IAU_FAL101 (GenBank accession number: MW845680) in (www.ncbi.nlm.nih.gov) with lysis range of ++ was sensitive to the phage, and plaque formation by it on the bacterial culture showed bacterial lysis (Table 4).

***The effect of different pH values and temperatures on bacteriophage stability***

The results of the effect of different pH values and temperatures on the stability of the phage Pɸ-Bw-Ab showed that the phage had the highest stability and antibacterial activity at pH=7. The lytic efficacy of the phage was significantly reduced at pH=4, 5, and 10 (Figure 4A). The results from the study of the effect of different temperatures on the stability of the phage Pɸ-Bw-Ab showed that this lytic phage had the highest stability (>90%) at the temperatures between 15 to 20 ^°^C. As the temperature increased, the stability of the phage decreased so that at 42 ^°^C, the phage stability percentage decreased down to approximately 40% (Figure 4B).

***Determining the rate of bacteriophage absorption***

The process of phage Pɸ-Bw-Ab adsorption on the surface of *A. baumannii* was evaluated. Phage lysates showed an increase in absorption of above 80% during 10 to 15 min, and the absorption rate was constant during the following 15 to 30 min incubation. The isolated phage had a rapid and ascending absorption rate relative to bacterial host cells in the first 5 to 10 min. On the other hand, approximately 20% to 25% of the phages were not absorbed by the host cell. The phage absorption rate is shown in Figure 4C.

***Bacteriophage one-stage growth curve***

In the one-step growth curve that was plotted to study the growth pattern and lytic activity of the phage Pɸ-Bw-Ab, the latency period was about 50 min and the burst size, after lysis of a single bacterial cell, was between 43 and 95 virions per cell. It means, after a 50-min latency phase, the cell explosion occurred between 50 and 60 min after infection. Finally, almost constant growth was observed from 60 to 130 min (Figure 4D).

***The bacteriolytic ability of P*** ***ɸ*** ***-Bw-Ab in MOIs***

The results from the phage activity in different MOI showed that in the control sample (MOI=0) bacterial growth was increased for 13 hr due to the absence of bacteriophage. At MOI=0.1 and MOI=1, the bacterial growth was continued for 10 hr. At MOI =10, due to the high phage titer (10 phages per bacterium), the bacterial growth rate had less increase than other MOIs in the first hour and was stopped at 9 hr. Phage amplification rates at different MOIs, based on the medium ODs are shown in Figure 6b. As shown, in all four MOIs the culture turbidity was close to OD=0.3 among the first 2 hours (Figure 4E).

***Results of restriction map and protein profile of isolated bacteriophage***

The results of restricted digestion patterns are shown in Figure 5A. Comparison of enzyme digestion patterns (enzyme cleavage) with each other showed that Enzyme digestion results showed that the isolated phage genome was sensitive to most enzymes and produced fragments of digestion pattern. Restriction enzymes pattern showed that phage nucleic acid had more cleavages by *HindIII *than other enzymes, and *HaeIII* had no effect on the phage gene. The partial digest is obtained from enzymatic digestion. The results of phage protein profiles are shown in Figure 5B according to a 10 to 180 KDa protein marker. By protein profile analysis in SDS-PAGE, it was estimated that the phage Pɸ-Bw-Ab had 43 to 90 kDa.

## Discussion

*A. baumannii*, *E. coli*, and *K. *pneumoniae were the most common gram-negative bacteria that caused burn wound infections in hospitalized patients. The results of the study showed a significant extent of resistance to various antibiotics among bacterial strains isolated from patient wounds. The common feature of the three main antibiotic-resistant bacteria was that they were sensitive to colistin. In a study carbapenem-resistant *A. baumannii* was detected in the patient burn wounds and high colistin sensitivity (99.9%) was observed among carbapenem-resistant *A. baumannii* (CRAB) strains which is consistent with the susceptibility of XDR strain of *A. baumannii* to colistin in the present study (54).

In the present study, the potential of the bacteriophage Pɸ-Bw-Ab which was isolated and identified from the inlet sewage of Isfahan treatment plant in Iran was determined for the removal of antibiotic-resistant bacteria. This bacteriophage was selected as the best candidate against *A.* *baumannii* strain IAU_FAL101. Phage identification results showed that this phage was a tailed virus with a dsDNA genome and belonged to the* Siphoviridae* family. The bacteriophage had the highest percentage of stability and antibacterial activity at pH 7 and at the temperature range between 15 to 20 ^°^C with a strong antibacterial potential to lyse bacterial cells. Bacteriophage Pɸ-Bw-Ab had a large burst size, thermal and pH stability, and high adsorption rate to the host cells in the first 5 to 10 min.

A group (2019) studied the phage Βϕ-R2096 which was used to control carbapenem-resistant *A. baumannii*. The phage belonged to the family *Myoviridae* and showed high bacteriolytic activity at MOI = 10. Our results demonstrated that the phage Pɸ-Bw-Ab belonged to the family *Siphoviridae* and showed high bacteriolytic activity at MOI=10 (55).

Furthermore, another study (2016) identified phage vB-GEC_Ab-M-G7 as phage *Myoviridae*. The phage vB-GEC_Ab-M-G7 had a short latent period and large burst size, wide host range, and thermal and pH consistency. Also, None of the eight (*BamHI, EcoR I, EcoRV, HindIII, HincII, PstI, DpnI, *and* SpeI*) restriction endonucleases used in this study digested phage vB-GEC_Ab-M-G7 (56). While in our research, bacteriophage Pɸ-Bw-Ab was identified as *Siphoviridae *and had a large burst size and viability. Among six (*HindIII, EcoRI, BamHI, KpnI, HaeIII, *and* XhoI)* restriction enzymes, phage nucleic acid had more cleavages by *HindIII *than other enzymes, and Phage Pɸ-Bw-Ab was not sensitive to *HaeIII* enzyme.

Researchers (2012) tested the impact of phage ɸkm18p that was isolated from sewage on XDR strains of *A.* *baumannii*, and they found that the phage effectively lysed the bacteria. The phage particle virion proteins were separated by SDS-PAGE. The most abundant protein was 39 kDa. The isolated phage belonged to the *Podoviridae* family and *HincII* and *NheI* enzymes digested phage ɸkm18p (57), but in our study, the isolated phage belonged to the *Siphoviridae *family, protein profile analysis was estimated 43 to 90 kDa and the most abundant protein was 43 kDa.

Popova *et al.* (2012) characterized the lytic bacteriophage AP22 infecting *A. baumannii.* The bacteriophage AP22 is classified as a member of the *Myoviridae* family. The phage AP22 exhibited rapid adsorption, a large burst size, and stability at various pH. The phage genome is digested with restriction endonucleases *HindIII, DraI, VspI, SspI, TaqI, AluI, RsaI, HinfI, MspI, CfrI, and EcoRI*. It is partially digested with *EcoRV, PstI, SalI, XmiI, SmiI, ClaI, BamHI, PvuII, BglII, EcoR91I, NcoI, *and* NheI* (58). In our study, phage was partially digested with* HindIII, EcoRI, BamHI, KpnI, *and* Xho *enzymes. 

A study (2020) identified phage vB_AbaP_D2. A one-step growth curve reflects the latent period, burst size, and release period. The results showed, the latent and rising period of phage vB_AbaP_D2 lasted for 20 and 30 min, respectively, and the average burst size (the number of phage particles released by each infected host cell) was 80±6 (59). Our research showed the latency period was about 50 min, after a 50-min latency phase, the cell explosion occurred between 50 and 60 min after infection. Finally, almost constant growth was observed from 60 to 130 min.

Yang *et al*. (2010) isolated Phage AB1 of *A. baumannii*. Restriction analysis indicated that phage AB1 was a dsDNA virus and had an icosahedral head with a non-contractile tail and whisker structures, and was classified as a member of the *Siphoviridae* family. The proteomic pattern of phage AB1, generated by SDS-PAGE, showed five major bands with molecular weight ranging from 14 to 80 kDa. Phage genomic DNA was digested with *Eco* RI**, ***Xba* I**, ***Bgl* II**, ***Bgl* II**/***Xba* I**,  ***Eco* RI/*Bgl* II, and *Eco* RI/*Xba* I enzymes (60). In our study, we used (*HindIII, EcoRI, BamHI, KpnI, HaeIII, *and* XhoI)* restriction enzymes and SDS-PAGE results showed major bands with molecular weight ranging from 43 to 90 kDa.

## Conclusion

*A. baumannii* has become a prevalent antibiotic-resistant bacterium in the special burn wards of hospitals. Due to the limitations of the effective spectrum of antibiotics in recent decades due to widespread drug resistance, phages with their specific properties can be considered as some of the most desirable and appropriate options to replace various antibiotics. Phages with the ability to replace antibiotics and control serious nosocomial infections can enter the treatment drugs as inexpensive antibacterial agents. It is hoped that by conducting various studies and evaluating the effectiveness of phages, they can be used as effective therapeutic agents against different antibiotic-resistant strains which may reduce the prevalence of infections caused by multidrug-resistant pathogens. Based on our study, the phage Pɸ-Bw-Ab had an antibacterial impact on other used bacterial spp, and also strong antibacterial effects and also strong antibacterial effects on Acinetobacter baumannii strains especially Acinetobacter baumannii strain IAU_FAL101, phage therapy would be an applicable proxy for antibiotics, especially in cases where there is resistance to antibiotics. 

## Authors’ Contributions

L RT, M D, NS N, and R M study conception and design; L RT data processing, collection, performing experiments, and draft manuscript preparation; L RT, M D, and NS N analysis and interpretation of results; M D, NS N, and R M critical revision of the paper; M D supervision of the research; L RT, M D, NS N, and R M final approval of the version to be published.

## Ethical Approval

This study was approved by the Ethics Committee of Islamic Azad University, Falavarjan Branch, Isfahan, Iran (code of ethics: IR.IAU.FALA.REC.1400.017).

## Conflicts of Interest

The authors declare that there are no conflicts of interest.
